# Crossing Borders, Missing Data - Cancer Inequities in Displaced and Migrant Populations: A Scoping Review

**DOI:** 10.3389/ijph.2026.1608687

**Published:** 2026-04-13

**Authors:** Brandon M. Godinich, Jourdyn Horton, Madeline Rodriguez, Anika Patel, Jessica Chacon

**Affiliations:** Department of Medical Education, Paul L. Foster School of Medicine, Texas Tech Health Sciences Center El Paso, El Paso, TX, United States

**Keywords:** cancer disparities, displaced populations, epidemiology, migrant health, U.S.-Mexico border

## Abstract

**Objectives:**

To review recent developments in cancer disparities affecting displaced and migrant populations in the U.S.–Mexico border region and identify barriers in public health and epidemiologic tracking.

**Methods:**

A structured scoping review was conducted using peer-reviewed studies and public health data published between 2010 and 2024. Sources included PubMed and governmental and binational public health reports. Inclusion criteria focused on border-specific, cancer-related evidence involving migrant and structurally vulnerable populations.

**Results:**

Cancer inequities were commonly associated with limited screening access, underinsurance, fragmented care, and binational surveillance gaps. Environmental and occupational exposures were identified as potential risk factors in border communities. Language barriers and mistrust of health systems limited engagement in preventive services, treatment continuity, and research participation.

**Conclusion:**

Cancer inequities among migrant populations in the U.S.–Mexico border region are closely linked to structural and policy barriers and gaps in cross-border epidemiologic infrastructure. Improved binational surveillance, culturally responsive outreach, and expanded access to screening and timely care may support more equitable cancer control.

## Introduction

The 1,954-mile U.S.-Mexico border represents a unique intersection of migration, health system fragmentation, and epidemiologic complexity. With more than 15 million residents living in the border region and substantial population mobility across the border each year, cancer control efforts encounter considerable challenges. Displaced and migrant populations include recent migrants, undocumented individuals, seasonal workers, and cross-border patients. These groups may face elevated cancer risk and less favorable cancer outcomes associated with fragmented access to preventive services and treatment, social vulnerability, and limited data capture within surveillance systems [1]. Cross-border utilization of oncology care further complicates continuity of care and outcome tracking cross jurisdictions [2].

Recent years have also seen heightened migration levels, with over 2.47 million southwest land border encounters reported in FY2023 [3]. This increased mobility may place additional pressure on resource-limited healthcare systems in the border counties and may contribute to pre-existing disparities in insurance, access to preventive services, and diagnostic timeliness. Compounding factors include legal precarity, fear of deportation, language barriers, and transient living conditions [4]. Some institutional, advocacy, and media reports have suggested that migrant safety and health can be affected by border enforcement practices and limited access to services [5].

This review synthesizes literature and public health data on cancer inequities faced by displaced and migrant populations along the U.S.-Mexico border, with emphasis on surveillance challenges, healthcare access barriers, and policy gaps. The goal is to identify evidence-informed considerations for public health planning and epidemiologic improvement in this binational region.

## Methods

### Study Design and Search Strategy

This structured scoping review followed the Arksey and O-Malley framework and was reported in accordance with the PRISMA-ScR guidelines. The objective was to map the breadth of literature examining cancer inequities faced by displaced and migrant populations along the U.S.-Mexico border region, with emphasis on surveillance limitations, access barriers, and structural determinants.

For this review, “displaced and migrant populations” were operationally defined as individuals residing in, transitioning through, or receiving care within the U.S.-Mexico border region who experience mobility, legal precarity, cross-border healthcare utilization, or migration-linked structural vulnerability. This included undocumented migrants, asylum seekers or forcibly displaced persons, seasonal and agricultural workers, cross-border patients receiving care in both countries, and hispanic residents of border counties experiencing migration-linked barriers. Studies focusing solely on nationally representative Hispanic populations without border-region stratification were categorized as contextual rather than border-specific.

A comprehensive search strategy was developed using medical subject heading (MeSH) terms and terms used in prior migrant-health systematic reviews. Databases searched included PubMed/Medline, Scopus and ScienceDirect. Gray literature was identified from the Centers for Disease Control and Prevention (CDC), U.S. Customs and Border protection (CBP), U.S.-Mexico Border Health Commission and the U.S. Department of Health and Human Services. Searches covered January 1, 2010 through April 30, 2024 (Tables 1,2).

**TABLE 1:** Characteristics of Included Sources by Cancer Focus and Primary Outcomes (U.S.-Mexico border region, 2010–2024).

Author (Year)	Study design	Cancer focus	Primary Outcome(s)	Key barriers identified	Surveillance/registry implications
de Heer [1]	Quantitative observational	Multiple cancers	Access to care; comorbidities	Underinsurance; language barriers; limited access to preventive care	Limited documentation of migrant status in registries
LaPelusa [2]	Quantitative cross-sectional survey	Multiple cancers	Cross-border utilization of oncology services	Fragmented care continuity; lack of medical record transfer; mobility	Incomplete cross-national tracking of cancer cases
Thompson [9]	Community-based intervention study	Cervical cancer	Pap smear uptake	Low baseline screening; cultural barriers; insurance gaps	Screening adherence inconsistently captured in regional databases
Carrillo [8]	Polic and environmental health analysis	Multiple cancers	Environmental carcinogen exposure	Air pollution; industrial exposure; binational environmental regulation gaps	Lack of harmonized environmental health surveillance
Salinas [20]	Quantitative ecological study	Obesity-related cancers	Food insecurity; obesity risk	Poverty; limited food access; socioeconomic vulnerability	Limited integration of social determinants in cancer surveillance
Healthy Border (HHS) [26]	Government public health framework	Cancer prevention & Surveillance	Binational health objectives	Infrastructure gaps; funding inequities; coordination barriers	Identified need for interoperable registry systems
Cancer Journal – Pediatric Leukemia Equity [27]	Quantitative epidemiologic study	Acute lymphoblastic leukemia	Incidence and equity analysis	Ethnic disparity; geographic structural disadvantage	Survival tracking inconsistencies across counties
Health profile of Mexican migration lows [28]	Quantitative observational	Cancer screening & access	Healthcare utilization patterns	Legal precarity; lack of insurance; transient mobility	Absence from formal registry systems
All Survival Border study [29]	Quantitative survival analysis	Acute lymphoblastic leukemia	Survival outcomes	Border residency-associated delays; specialty access	Geographic survival disparities underreported
JCO Oncology practice [13]	Quantitative systems analysis	Multiple cancers	Quality of cancer prevention and treatment	Limited oncology specialists; delayed diagnosis; equipment shortages	Sparse oncology reporting infrastructure
Ethnic and Border Differences in Blood Cancer [6]	Quantitative population-based study	Hematologic malignancies	Presentation stage; survival	Geographic inequity; delayed presentation	Registry limitations in capturing migration history
Rural health information Hub – Border Health Status [25]	Government public health report	Physician workforce metrics; resource capacity	Physician workforce metrics; resource capacity	PCP shortages; limited oncology access; rurality	Infrastructure deficits impact cancer data completeness

**TABLE 2:** Prevalence of barrier themes (based only on 12 included sources) (U.S.-Mexico border region, 2010–2024).

Barrier theme	Number of supporting studies (n = 12)	Percent of total (%)	Evidence type
Insurance/financial barriers	6	50%	Quantitative + policy
Language/health literacy	4	33%	Quantitative
Legal precarity/mistrust	2	17%	Policy
Care fragmentation/mobility	4	33%	Quantitative
Screening infrastructure	5	42%	Quantitative
Environmental/occupational	3	25%	Quantitative
Clinical trial Barriers	1	8%	Quantitative
Registry/surveillance gaps	5	42%	Quantitative + policy
Policy/governance	4	33%	Policy

Search strategies were adapted for each database. The PubMed search string was:

(“cancer”[MeSH Terms] OR cancer* OR oncology OR malignant*) AND (“migrant*” OR “immigrant*” OR “refugee*” OR “displaced” OR “undocumented” OR “foreign-born”) AND (“U.S.-Mexico border” OR “US-Mexico border” OR “Mexico-U.S. border” OR “border health” OR “Texas border counties”) AND (“disparity*” OR “inequity*” OR “access” OR “screening” OR “epidemiology*” OR “registry”) Filters: English, 2010–2024.

Government and policy websites were searched using combinations of “cancer”, “border health”, “U.S.-Mexico border”, “migrant health”, “registry”, and “surveillance”.

### Eligibility Criteria

Studies were included if they met all of the following:

- Published between January 2010 and April 2024.
- Published in English.
- Examined cancer-related outcomes (incidence, screening, treatment, survival, survivorship, registry/surveillance).
- Focused on populations located in or directly linked to the U.S.-Mexico border region.
- Included migrant, displaced, cross-border, undocumented, or structurally vulnerable Hispanic populations.

Eligible study designs included observational quantitative studies, qualitative studies, mixed-methods studies, government or institutional public health reports and policy analyses directly related to border-region cancer epidemiology.

### Studies Were Excluded if They

- Were published prior to 2010.
- Did not focus on cancer-related outcomes,
- Examined general Hispanic populations without border-region stratification.
- Focused solely on migration statistics without cancer relevance.
- Were editorials without analytic or empirical content.
- Did not provide sufficient data to assess border-region relevance.

Media articles and historical reports prior to 2010 were excluded from the formal synthesis but may be referenced contextually in the discussion.

#### Study Selection and Data Processing

All identified records were imported into a reference management system, and deduplicated prior to screening. Two reviewers independently screened titles/abstracts, followed by full-text review. Discrepancies at any stage were resolved by consensus.

Data extraction used a standardized form capturing author/year, study design, cancer type/outcome focus, key barriers, and surveillance/registry implications. Studies were coded into predefined domains: insurance/financial barriers; screening and oncology infrastructure constraints; environmental/occupational exposures; clinical trial access barriers; registry/binational surveillance gaps; and broader policy/governance barriers.

**FIGURE 1:**
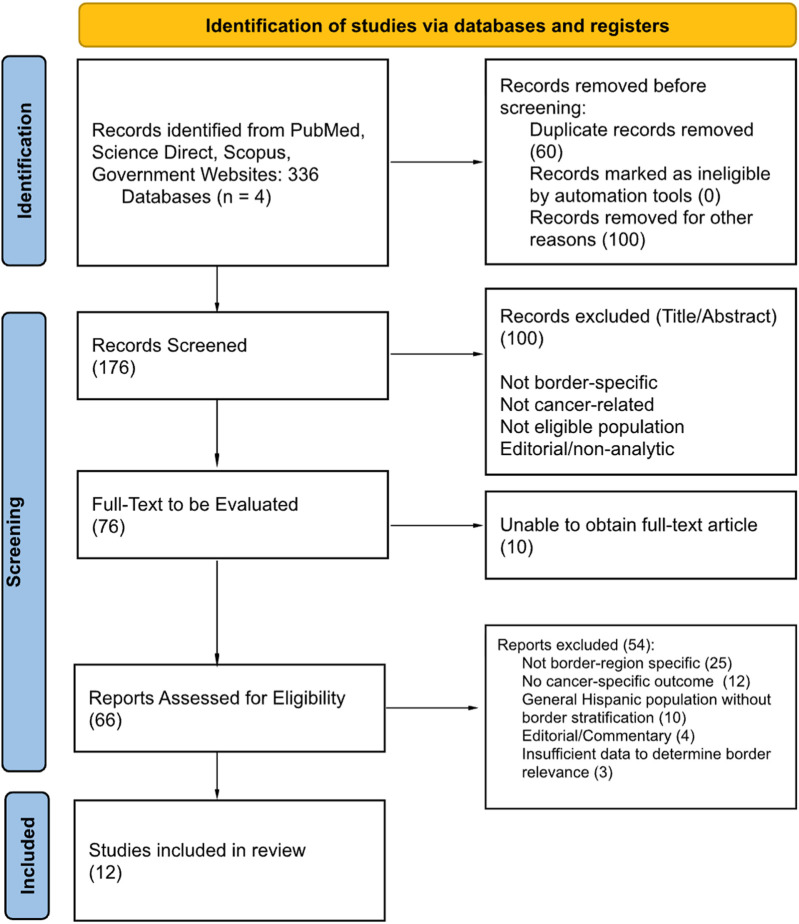
PRISMA study flowchart (U.S.-Mexico border region, 2010–2024).

Given heterogeneity in study design, methodology, and outcome reporting, meta-analysis was not appropriate. Findings were synthesized narratively, and theme frequency was summarized across included studies. Formal risk-of-bias scoring was not performed (consistent with scoping review methodology), but study design and key methodological limitations were recorded. The study selection process, including the number of records identified, screened, excluded with reasons, and included in the final synthesis, is summarized in Figure 1.

## Results

### Search Results and Study Characteristics

The database and gray literature search yielded 336 records. After removal of 160 duplicates, 176 records underwent title/abstract screening. 100 articles were excluded due to non-eligible population, setting, or lack of cancer-specific outcomes. Full-text articles were reviewed for the remaining 76 articles. A total of 12 full-text articles met all inclusion criteria and were included in the final synthesis.

The included sources represented a mix of quantitative observational studies and border-relevant institutional/governmental reports. Most studies focused on Hispanic border residents, cross-border patients, and migrant populations experiencing structural vulnerability in U.S. border counties and adjacent Northern Mexican regions. The focus on cancer types and themes spanned cervical cancer screening, hematologic malignancies, infection-associated cancers, health system capacity and surveillance infrastructure.

### Population Representation

Across included studies, study populations were predominantly Hispanic residents of Texas border counties and cross-border patients receiving care in both the United States and Mexico [1, 2, 6]. Migrant populations traversing the northern Mexican border were characterized by high mobility, limited continuity of care, and inconsistent access to preventive services [7]. Although displaced and migrant populations were consistently described as structurally vulnerable, documentation status and forced displacement were rarely measured directly. Most studies relied on geographic residence, cross-border utilization patterns, or ethnicity-based proxies [8].

### Barriers to Cancer Screening and Early Detection

**FIGURE 2:**
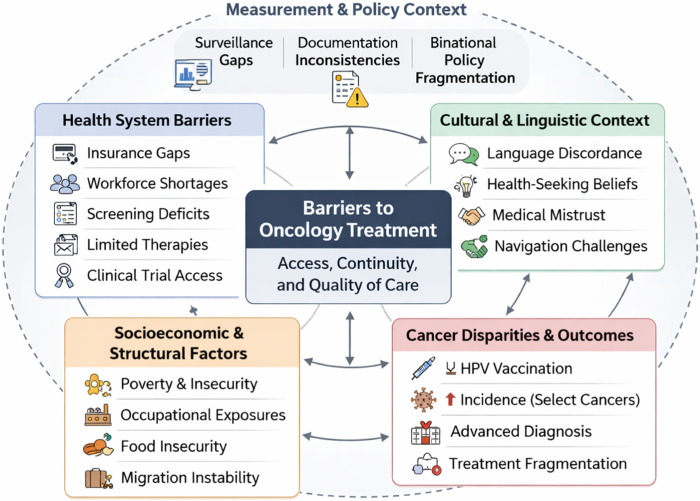
Interacting multilevel domains influencing oncology treatment in migrant and displaced populations (U.S.-Mexico border region, 2010–2024).

Barriers to screening and early detection were most commonly linked to insurance and affordability constraints and to limited screening capacity in border health systems (Figure 2). Among Hispanic women in the border region, breast cancer screening was reported at 56% compared with 72% among non-Hispanic white women nationally [9]. Cervical cancer screening rates among women aged 21–65 in certain Texas border counties were reported as low as 52% [10]. HPV vaccination completion represented an additional preventive gap, with only 44% of Hispanic women completing the vaccine series compared with 62% of non-Hispanic women [11]. These disparities were reported alongside high uninsured rates, which exceed 30% in some border counties [1].

Promotora -led interventions were associated with improved screening adherence among previously non-compliant women, with more than 75% completing pap smear screening following outreach [9]. Mobility further disrupted continuity of screening among migrants lacking records and consistent access to preventive services [7].

### Healthcare Infrastructure and Specialist Access

Border-region healthcare infrastructure constraints were consistently reported, including workforce shortages and limited oncology specialty access. In some Texas border counties, the primary care physician-to-population ratio was approximately 1 per 1,957 residents compared with a national average of about 1 per 1,320 [12]. Multiple sources described limited specialist availability and delayed diagnostic pathways, with prolonged time (more than 90 days) from symptom onset to diagnosis reported in border communities [13]. Constraints in screening equipment availability were also noted in high-need counties. For example, Hidalgo County has one mammogram machine per 26,000 women over 40 years old [10]. Furthermore, the lack of bilingual staff and language discordance within clinical encounters was described as contributing to delayed care and fragmented communication [14].

#### Cancer Incidence and Survival Inequities

Infection-associated cancers were reported to be disproportionately elevated among Hispanic populations. Between 2014 and 2018, liver and intrahepatic bile duct cancer incidence was 20.3 per 100,000 among Hispanics compared with 10.9 per 100,000 among non-Hispanic Whites (IRR = 1.86) [11]. Gallbladder cancer incidence was 2.5 per 100,000 versus 1.4 (IRR = 1.79), and stomach cancer incidence was 8.9 versus 4.7 (IRR = 1.89) [11].

Pediatric acute lymphoblastic leukemia was reported to occur at approximately 1.5 times the rate among Hispanic children compared with non-Hispanic populations [15]. Texas-specific analyses demonstrate that children residing in border counties experienced lower leukemia survival outcomes independent of ethnicity [6]. Cervical cancer mortality among Hispanic women has been reported as approximately 40% higher than among non-Hispanic White women [9].

### Environmental and Occupational Exposures

Environmental and occupational exposures were recurrently discussed as potential contributors to cancer risk in border communities, particularly air pollution and pesticide exposure. El Paso and Laredo ranked among the highest Texas regions for ozone and PM2.5 exposure levels [8]. Respiratory assessments in border populations have documented elevated asthma prevalence associated with air pollution exposure [16]. Additionally, water contamination was reported in more than 20% of sampled sites, with elevated nitrate levels associated with gastrointestinal cancer risk [17]. Occupational pesticide exposure among migrant and agricultural workers was associated with a 2.6-fold increased risk of hematologic malignancies [18]. A case series in Texas also reported pediatric poisonings from agricultural pesticide exposure [19].

Among maquiladora workers in northern Mexico, prolonged solvent exposure has been associated with increased bladder and liver cancer incidence. Food insecurity was also reported as prevalent in some border households (32%), representing a structural determinant potentially associated with obesity-related cancer risk [20]. Although these exposures were consistently identified, quantification varied substantially across sources.

Undocumented individuals and structurally vulnerable migrants were frequently represented in institutional and policy analyses examining chronic disease burden and access barriers in the border region [21]. Seasonal and agricultural workers were identified as a high-risk subgroup due to occupational carcinogen exposure and reduced healthcare engagement [18].

### Clinical Trial Access

Clinical research participation barriers were described in terms of underrepresentation, limited research infrastructure, and mistrust. Only 3% of cancer clinical trial participants in the U.S. identify as Hispanic, despite Hispanics comprising 19% of the national population [22]. It was reported that low participation rates were attributed in part to limited research infrastructure and bilingual recruitment efforts [23]. In a qualitative study, Mexican-American respondents cited fear of experimentation, lack of understanding, and transportation difficulties as key deterrents to clinical trial participation [14]. A recent policy analysis also noted that funding for border-region clinical research is 5 times lower *per capita* than in urban U.S. regions [23].

### Migration Pressures and Care Disruption

Migration across the U.S.–Mexico border reached 2.475 million encounters in fiscal year 2023 compared with 977,000 in fiscal year 2019 [3]. This surge was reported to place additional strain on already resource-limited border clinics and contribute to disruptions in continuity of care [2]. Migrant patients frequently arrive without complete medical records or consistent screening histories, complicating cancer detection and follow-up [7]. Mobile patients are also less likely to engage in preventive services, with screening rates up to 40% lower than the regional average [9]. The logistical burden includes increased wait times and shortages of vaccines and testing kits [10]. Community health workers in the region report increasing workload intensity and burnout under these pressures [12].

### Binational Surveillance and Registry Limitations

Binational epidemiologic surveillance limitations were dominant in border health reports and policy analyses. Displaced persons, particularly migrants and seasonal workers, often fall outside the scope of national cancer registries due to lack of a permanent address or documentation [21]. The absence of standardized data collection tools and interoperable platforms between the U.S. and Mexico was described as resulting in in fragmented reporting [8]. For example, cancer cases diagnosed in Mexico but treated in U.S. clinics are rarely tracked systematically in either country [2]. Moreover, there is no shared patient identifier across border systems, complicating continuity of care and follow-up [24]. In some regions, over 40% of patients receive care in both countries but are counted only in one registry, which may contribute to underreporting [25]. Limited cross-agency collaboration and incompatible definitions of health indicators further impair real-time monitoring of incidence and mortality trends. Public health initiatives such as Healthy Border 2030 have emphasized improved binational data coordination, yet operational and political barriers have been described as limiting real-time surveillance integration [26].

### Relative Weight of Evidence Across Thematic Domains

Across the 12 included sources, insurance and financial barriers were among the most frequently cited determinants of inequitable cancer outcomes in border communities, followed by language barriers/reduced health literacy, care fragmentation/mobility and policy/governance. Screening infrastructure limitations, including equipment shortages and limited preventive capacity, were regularly documented in clinical and public health analyses [10]. Workforce shortages and constrained oncology specialist access were also recurrent themes in institutional assessments of border-region health systems [12].

Environmental and occupational carcinogen exposure emerged as a distinct but less frequently quantified domain, primarily supported by observational and environmental health reports [8]. Clinical trial access disparities were addressed in a smaller subset of qualitative and policy-focused studies examining participation patterns among Mexican-American populations [22].

Binational surveillance fragmentation and registry underrepresentation were repeatedly identified in governmental and policy documents as substantial epidemiologic gaps that may limit accurate cancer burden estimation [26]. Overall, the evidence base is weighted toward observational and descriptive analyses, with comparatively fewer interventional or longitudinal studies directly evaluating outcomes among displaced border populations [1].

## Discussion

This scoping review highlights themes in cancer inequities on the U.S.-Mexico border region. These inequities are not solely attributable to isolated access gaps, but appear consistent with layered structural disadvantage operating within a fragmented binational health environment [1]. Although the included studies varied in design and scope, the convergence of findings across domains is consistent with the interpretation that cancer disparities in displaced and migrant populations are structurally patterned rather than incidental. The limited number of interventional or longitudinal studies further indicates that the region has been more extensively described than meaningfully transformed [1]. The analytical challenge, therefore, is not identifying disparities, but understanding how overlapping forces may interact to reinforce them.

The population data illustrate a central paradox that the communities most frequently described as structurally vulnerable were rarely directly measured as displaced or undocumented [8]. By relying on geographic or ethnic proxies, the literature implicitly acknowledges the presence of forced migration and documentation precarity while simultaneously rendering them statistically indistinct. This creates an epidemiologic blind spot in which structural vulnerability is recognized conceptually but not operationalized analytically. As a result, cancer burden may be underestimated among populations whose mobility and legal status most directly disrupt preventive care. The absence of direct measurement can itself be interpreted as a structural finding.

Screening inequities, while quantitatively clear, may reflect deeper systemic constraints. Lower breast and cervical cancer screening rates in border counties [10] and lower HPV vaccine completion among Hispanic women [11] are often framed as individual-level utilization gaps. However, when these data are interpreted alongside high uninsured rates [1] and documented screening infrastructure shortages [10], they are consistent with a supply-demand imbalance embedded within regional policy and funding structures. The success of promotora-led interventions demonstrates that when culturally concordant outreach is paired with access, engagement improves [9]. This implies that “non-compliance” may at times be attributed to patient behavior rather than structural design. Yet the scalability challenge underscores the broader idea that localized interventions can mitigate inequity, but they may be insufficient to offset chronic underinvestment in border health systems.

Healthcare infrastructure constraints further illuminate how delayed diagnoses may become normalized within resource-limited settings. Workforce shortages [12], prolonged diagnostic intervals [13], and equipment scarcity [10] collectively create conditions in which timeliness may be compromised. These delays may contribute to the higher cervical cancer mortality reported among Hispanic women [9] and the worse leukemia survival outcomes observed in Texas border counties [6]. Importantly, these survival disparities persist even when ethnicity is accounted for, suggesting that place-based resource allocation may exert an independent influence [6]. Geography, in this context, may function as a determinant of survival.

The elevated incidence of infection-associated cancers among Hispanic populations appears intertwined with the preventive gaps and environmental exposures identified in the review [11]. While biologic susceptibility, is sometimes invoked in discussions of disparity, the clustering of infection-related malignancies in underinsured and resource-constrained communities is consistent with the influence of upstream determinants such as limited vaccination, delayed treatment of chronic infections, and constrained specialty follow-up. The analytic implication is that incidence patterns may reflect limitations in primary and secondary prevention infrastructure rather than immutable risk.

Environmental and occupational exposures add another dimension of cumulative vulnerability. Reports of elevated air polluation [8], nitrate contamination [17], and increased hematologic malignancy risk among pesticide-exposed workers [18] suggest that cancer risk in border communities may be shaped by labor patterns and industrial geography. These exposures disproportionately affect migrants, agricultural workers, and maquiladora employees who simultaneously face reduced healthcare engagement. The interaction between environmental carcinogen exposure and limited screening capacity may contribute to later-stage presentation. However, the variability in exposure quantification indicates that environmental health surveillance may be underdeveloped relative to the scale of documented risk [8]. The absence of standardized exposure metrics could obscure dose-response relationships critical for targeted mitigation.

Clinical trial underrepresentation further reflects structural exclusion from innovation pathways. The discrepancy between Hispanic population proportion and trial participation suggests not merely a recruitment issue but potential infrastructure limitations [22]. When *per capita* research funding in border regions is substantially lower than in urban centers, participation barriers may reflect institutional constraints in addition to cultural factors [23]. Qualitative accounts of mistrust and transportation barriers must therefore be interpreted within a context of geographic and financial marginzaliation [14]. Without embedded research infrastructure, disparities in clinical trail access May persist despite outreach efforts.

Migration pressures may amplify existing system limitations rather than create them Independently. The surge in border encounters in recent years [3] exposes the fragility of already strained clinics and workforce systems [12]. Mobile patients’ lower screenings rates [9] and lack of transferable medical records [7] reveal how continuity of care remains territorially bound in a population that is not. The analytic issue is not mobility itself, but the absence of interoperable systems capable of accommodating mobility. Health systems structured around fixed residency may be less adaptable to populations defined by movement.

Perhaps the most consequential structural finding is the fragmentation of binational surveillance. When migrants and cross-border patients are excluded from registries [21], and no shared identifier exists across systems [24], cancer burden may become administratively partitioned rather than clinically coherent. The reported undercounting of patients receiving care in both countries suggests that incidence and survival metrics may be affected by measurement limitations [25]. Policy initiatives such as Healthy Border 2030 acknowledge the need for coordination, yet operational barriers persist [26]. Without harmonized data standards and cross-border interoperability, even well-designed interventions may be difficult to evaluate comprehensively.

Across domains, insurance and financial barriers were most frequently cited, but the analytic synthesis suggests that insurance expansion alone ay be insufficient to resolve inequities. Workforce capacity [12], equipment distribution [10], environmental mitigation [8], research infrastructure [22], and surveillance intergration [26] operate as interdependent determinants. The border region can be conceptualized as a transnational ecosystem in which health policy, labor markets, migration flows and environmental regulation intersect. Cancer disparities in displaced populations therefore appear to reflect the cumulative effects of fragmented governance and structural constraint.

Future research may benefit from moving beyond descriptive disparity documentation and toward implementation-focused and longitudinal evaluation. Given the documented mobility of border populations and the fragmentation of registry capture, cross-border cohort studies could improve the understanding of cancer outcomes in mobile patients. Similarly, the variability in reported environmental and occupational exposures suggests that standardized, regionally coordinated measurement frameworks may strengthen risk estimation and comparability across jurisdictions. The recurrent identification of binational surveillance fragmentation in governmental and institutional reports indicates that greater data interoperability and collaborative data exchange mechanisms could enhance burden estimation and program evaluation. Without improved alignment between mobility patterns and surveillance infrastructure, accurate measurement of cancer outcomes in displaced populations may remain limited, potentially constraining preventive and early detection efforts.

### Limitations

This review has several limitations that warrant consideration. The available evidence base is predominantly observational, descriptive, or qualitative, which may limit causal inference regarding the relationship between migration-related structural factors and cancer outcomes. Few studies employed longitudinal designs or evaluated interventions, and most did not directly measure documentation status, forced displacement, duration of migration, or patterns of cross-border mobility. Instead, many relied on geographic residence, cross-border care utilization, or ethnicity-based proxies to approximate structural vulnerability. While informative, these proxies may underestimate heterogeneity within migrant and displaced populations and limit the precision of comparisons across studies.

In addition, although gray literature searches were structured and targeted, they may have not been fully exhaustive, and some institutional or policy reports were included to provide contextual framing of infrastructure and surveillance gaps. Variability in methodological rigor across sources may influence the strength of certain conclusions. Finally, heterogeneity in study design, populations, and reported outcomes precluded quantitative meta-analysis. As a result, synthesis relied on narrative integration and thematic prevalence mapping rather than pooled effect estimates, and findings should therefore be interpreted as hypothesis-generating rather than definitive.

### Conclusion

Cancer inequities among displaced and migrant populations on the U.S.-Mexico border appear to reflect the cumulative effects of structural vulnerability, constrained health system capacity, environmental and occupational risk, research underrepresentation, and fragmented binational surveillance. The evidence synthesized in this review suggests that disparities in screening, stage at diagnosis, survival, and clinical trial participation are not isolated phenomena but are situated within financing models, workforce distribution, mobility-related care disruption, and incomplete cross-border data systems. Although the current literature is largely descriptive, it consistently points to potentially modifiable structural determinants, including insurance gaps, specialist shortages, environmental exposures, and registry fragmentation. Addressing cancer disparities in this region will likely depend on coordinated binational coordination, policy and administrative alignment, investment in preventive and oncology infrastructure, rigorous evaluation of scalable community-based interventions, improved research inclusion, and interoperable surveillance systems capable of capturing mobile populations. Without structural reform that recognizes mobility and transnational care as defining features of the border context, cancer control efforts may remain constrained and inequities may persist.
